# Predicting postoperative neurological outcomes of degenerative cervical myelopathy based on machine learning

**DOI:** 10.3389/fbioe.2025.1529545

**Published:** 2025-03-04

**Authors:** Shuai Zhou, Zexiang Liu, Haoge Huang, Hanxu Xi, Xiao Fan, Yanbin Zhao, Xin Chen, Yinze Diao, Yu Sun, Hong Ji, Feifei Zhou

**Affiliations:** ^1^ Department of Orthopaedics, Peking University Third Hospital, Beijing, China; ^2^ Engineering Research Center of Bone and Joint Precision Medicine, Ministry of Education, Beijing, China; ^3^ Beijing Key Laboratory of Spinal Disease Research, Beijing, China; ^4^ Department of Orthopaedics, China Emergency General Hospital, Beijing, China; ^5^ Information Management and Big Data Center, Peking University Third Hospital, Beijing, China

**Keywords:** degenerative cervical myelopathy, spine surgery, machine learning, outcome, prediction model

## Abstract

**Introduction:**

This study aimed to develop machine learning models to predict neurological outcomes in patients with degenerative cervical myelopathy (DCM) after surgical decompression and identify key factors that contribute to a better outcome, providing a reference for patient consultation and surgical decision-making.

**Methods:**

This retrospective study reviewed 1,895 patients who underwent cervical decompression surgery for DCM at Peking University Third Hospital from 2011 to 2020, with 672 patients included in the final analysis. Five machine learning methods, namely, linear regression (LR), support vector machines (SVM), random forest (RF), XGBoost, and Light Gradient Boosting Machine (LightGBM), were used to predict whether patients achieved the minimal clinically important difference (MCID) in the improvement in the Japanese Orthopedic Association (JOA) score, which was based on basic information, symptoms, physical examination signs, intramedullary high signals on T2-weighted (T2WI) magnetic resonance imaging (MRI), and various scale scores. After training and optimizing multiple ML algorithms, we generated a model with the highest area under the receiver operating characteristic curve (AUROC) to predict short-term outcomes following DCM surgery. We evaluated the importance of the features and created a feature-reduced model. The model’s performance was assessed using an external dataset.

**Results:**

The LightGBM algorithm performed the best in predicting short-term neurological outcomes in the testing dataset, achieving an AUROC value of 0.745 and an area under the precision*–*recall curve (AUPRC) value of 0.810. The important features influencing performance in the short-term model included the preoperative JOA score, age, SF-36-GH, SF-36-BP, and SF-36-PF. The feature-reduced LightGBM model, which achieved an AUROC value of 0.734, also showed favorable performance. Moreover, the feature-reduced model showed an AUROC value of 0.785 for predicting the MCID of postoperative JOA in the external dataset, which included 58 patients from other hospitals.

**Conclusion:**

We developed models based on machine learning to predict postoperative neurological outcomes. The LightGBM model presented the best predictive power regarding the surgical outcomes of DCM patients. Feature importance analysis revealed that variables, including age, preoperative JOA score, SF-36-PF, SF-36-GH, and SF-36-BP, were essential factors in the model. The feature-reduced LightGBM model, designed for ease of application, achieved nearly the same predictive power with fewer variables.

## 1 Introduction

Degenerative cervical myelopathy (DCM) is a progressive, non-traumatic degenerative disease that leads to the compression of the cervical spinal cord (Fehlings et al., 2013), resulting in the loss of manual dexterity; gait and balance disturbances; sensory loss in the hands or feet; arm or hand weakness; and defecatory or urinary frequency, urgency, or hesitancy (Okada et al., 2009). With the aging society, DCM has become an urgent clinical and public health concern (GBD, 2017 Disease and Injury Incidence and Prevalence Collaborators et al., 2018).

For moderate and severe DCM, as well as for patients with progressive disease, surgical decompression is recommended; it has been proved to improve neurological function and quality of life for patients. However, studies indicate that 5%–30% of patients did not achieve a satisfactory outcome after surgery (Fejer et al., 2006). In previous studies, the factors affecting surgical outcomes included age, baseline severity score, duration of preoperative symptoms, signs and symptoms, comorbidities, and high signal intensity (SI) on T2-weighted (T2-WI) magnetic resonance imaging (MRI) (Gembruch et al., 2021; Tetreault L. A. et al., 2015). However, predicting outcomes for individual patients is a multivariable and chaotic system of interactions. The influence of various variables and their interactions on postoperative outcomes cannot be accurately assessed by traditional statistical methods (Combi, 2017). The significance of different features in predicting surgical outcomes for patients with DCM remains undetermined (Tetreault et al., 2013; Tetreault et al., 2014; Tetreault et al., 2016).

Machine learning is a developing method that can be applied to clinical datasets for the purpose of developing robust risk models and redefining patient classes (Shah et al., 2023). Previous studies have used machine learning methods to establish models to predict postoperative neurological function in patients with better predictive power than traditional statistical models. These models have achieved good performance, with area under the receiver operating characteristic curve values (AUROC) ranging from 0.7 to 0.9 (Khan et al., 2021; Merali et al., 2019; Song et al., 2024). However, a large-scale study and prospective external validation are still lacking. A tradeoff exists in machine learning between model complexity and generalizability to new datasets. One solution is to build a model with fewer features and appropriate performance (Shah et al., 2023).

In this study, we aimed to develop machine learning models to predict individual DCM patients’ neurological outcomes after surgery and select the model with the best performance. Additionally, we aimed to identify influential features and create a model with fewer features and good performance in external validation, which would benefit the clinical practice of spine surgeons.

## 2 Materials and methods

### 2.1 Patient population

This study was approved by our hospital’s Medical Research Ethics Committee (LM2021299). This retrospective study reviewed 1,895 patients who underwent DCM surgical decompression, including laminoplasty, laminectomy and fusion, anterior cervical decompression and fusion, and anterior cervical corpectomy and fusion from 2011 to 2020 at Peking University Third Hospital. The data were obtained from the Electronic Data Capture (EDC) system at the Peking University Third Hospital Information Center. All private information was masked.

The inclusion criteria were as follows: 1) age ≥18; 2) diagnosed with DCM; 3) underwent surgical decompression for DCM including laminoplasty, laminectomy and fusion, anterior cervical decompression and fusion, and anterior cervical corpectomy and fusion; and 4) had at least one follow-up record between 3 and 6-month follow-up. Exclusion criteria included a history of spinal tumor, active infection, rheumatoid arthritis, cervical trauma, ankylosing spondylitis, and previous cervical spine surgery. Patients with missing scale scores and invalid follow-up records were also excluded. Patients with JOA scores ≥15 were excluded to avoid a ceiling effect as they could not achieve the minimal clinically important difference (MCID). We standardized surgical indications and approach selection based on guidelines and our cervical spine professional group’s recommendations. A total of 672 patients were included in the final analysis.

### 2.2 Baseline data and outcomes

The baseline data for the training models included demographics (age, sex, and profession), personal history (number of comorbidities, history of tobacco and alcohol intake, etc.), symptoms, signs, imaging examination (intramedullary high signals on T2WI MRI), and preoperative scale scores (JOA and SF-36). We applied a natural language processing (NLP) algorithm to extract symptom data from unstructured electronic medical records. Personal history, symptoms, and signs recorded as binary variables were selected based on the standardized medical records for orthopedics.

We examined patients’ JOA scores 3–6 months after surgery to evaluate neurological outcomes. The primary outcome was whether patients achieved MCID in JOA scores. According to previous research, the MCID for the JOA score in DCM patients was 2.5 (Maki et al., 2021). Categorical features such as professions were transferred to one-hot coding.

### 2.3 Data pre-processing

In the baseline dataset, all the demographic data were valid. Based on word segmentation of symptoms, signs, diseases, and other information using natural language processing technology, synonym information of the standard terminology database is introduced for standardization and level normalization. By “document category prediction” and “chapter prediction in the document,” different medical records and chapter contents are distinguished. Based on this, a combined approach using a bidirectional long short-term memory conditional random field (BiLSTM-CRF) network along with rule matching was employed to extract clinical data information. The NLP algorithm would leave blanks in the dataset if there were no matched descriptions in electronic medical history records. Therefore, we filled all the missing data in binary features (personal history, symptoms, and signs) with “normal” or “negative.”

### 2.4 Training machine learning models and performance evaluation

We randomly split the dataset into a training set (70%) and a test set (30%). Five machine learning algorithms, namely, linear regression (LR), support vector machine (SVM), random forest (RF), extreme gradient boosting (XGBoost), and light gradient boosting machine (LightGBM), were trained using 3-fold cross-validation on the training set with default hyperparameters. Model performance was evaluated using five metrics, namely, balanced accuracy, weighted precision, weighted recall, weighted area under the precision*–*recall curve (AUPRC, Supplementary Document), and area under the receiver operating characteristic curve (AUROC). Based on these metrics, we selected the optimal model for further refinement. We employed a bootstrap approach to obtain robust estimates and compute confidence intervals (CIs). Specifically, we repeated the cross-validation procedure 1,000 times with varied splits of the training data. The 95% CI for each metric was approximated using the 2.5th and 97.5th percentiles of the resulting performance distributions.

### 2.5 Hyperparameter optimization

Hyperparameter optimization was performed on the selected model using a grid search algorithm. We first predefined a set of candidate values for each hyperparameter and then exhaustively evaluated all combinations via 3-fold cross-validation on the training set. The optimal hyperparameter set was chosen based on the highest AUROC.

### 2.6 Testing set evaluation

Using the five aforementioned metrics, the fine-tuned model was subsequently evaluated on the test set. To calculate CIs, we again applied the bootstrap method by randomly splitting the dataset 1,000 times and retraining the model (with fixed best hyperparameters) on each new training split. The resulting performance distributions were used to derive the 95% CIs (2.5th and 97.5th percentiles).

All machine learning models were implemented in Python 3.8.5 using the scikit-learn, XGBoost, and LightGBM library. We adhered to the transparent reporting of multivariable prediction models for individual prognosis or diagnosis (TRIPOD) guidelines throughout model development.

### 2.7 Feature importance analysis

The machine learning module used to train the model offers built-in methods for assessing feature importance. For linear models (LR and SVM), importance is determined by the absolute value of the learned coefficients. For tree-based models (RF, XGBoost, and LightGBM), importance is calculated based on the frequency of each feature used for splitting nodes across all trees. Additionally, the Shapley additive explanation (SHAP) value of a feature is calculated by determining its marginal contribution across all possible feature subsets, and these contributions are then averaged to yield the feature’s overall impact on the prediction.

### 2.8 Feature-reduced model

To improve the model’s compatibility across different healthcare systems, we aimed to reduce the number of features. According to the importance of features in the final model and based on clinical experience, we selected a subset of features to build a feature-reduced machine learning model. We then extracted the relevant feature data from the previous training and testing sets to train this model. The outcome measures remained consistent with those aforementioned.

### 2.9 External model validation

Another dataset containing 31 patients from West China Hospital and 27 patients from Shanghai Changzheng Hospital was used as the external validation set. The dataset includes patients’ demographics, JOA scores, and SF-36 scores, which were used to validate the feature-reduced model. We analyzed AUROC values and decision curve analysis (DCA) to evaluate the performance of the feature-reduced model in the external dataset.

## 3 Results

### 3.1 Patients’ characteristics

**FIGURE 1:**
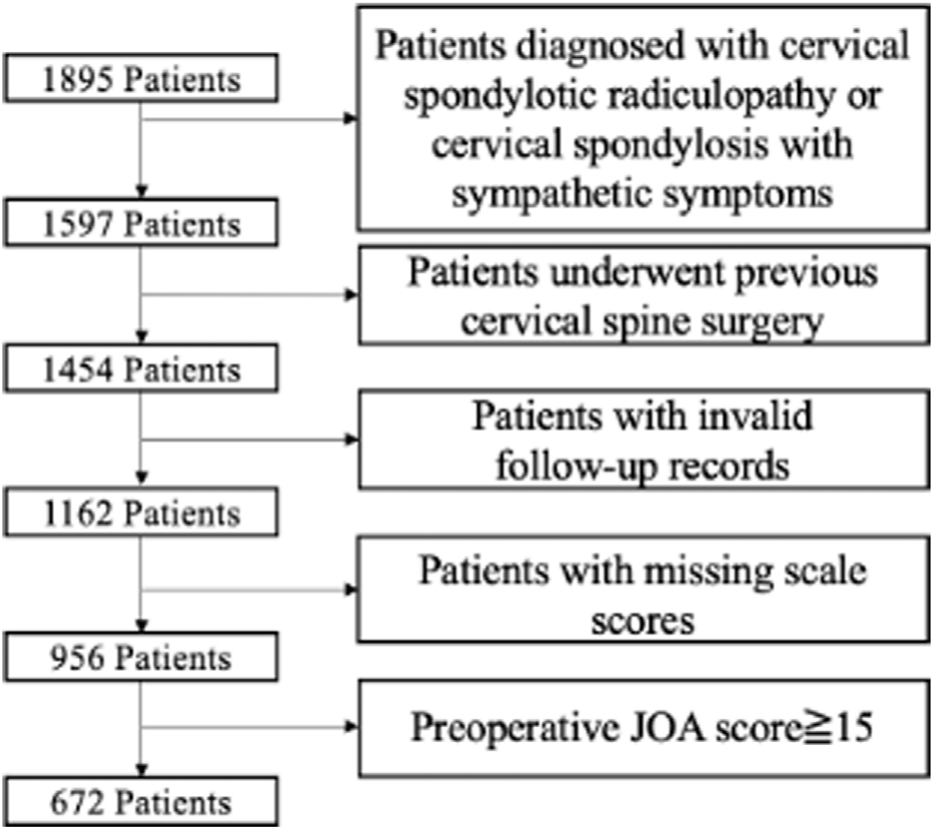
Patient selection flow chat.

Of the 1,895 patients, 298 were excluded because they were diagnosed with cervical spondylotic radiculopathy or cervical spondylosis with sympathetic symptoms; 143 patients were excluded due to previous cervical spine surgery history; 498 patients’ records were invalid or missing; and 284 patients had preoperative JOA scores ≥15. Finally, a dataset with 672 patients and 63 features was generated (Figure 1). The baseline characteristics are shown in Table 1. In addition, 369 patients reached the MCID of JOA score improvement at the short-term follow-up postoperatively.

**TABLE 1:** Baseline characteristics of the patients.

	Unimproved group (n = 303)	Improvement group (n = 369)	*P*-value
Age, M (IQR), years	55 (14)	53 (15)	0.116
SF-36-PF, M (IQR)	70 (35)	65 (35)	0.002*
SF-36-RP, M (IQR)	0 (25)	0 (0)	0.021*
SF-36-BP, M (IQR)	69 (36)	57 (35.5)	0.001*
SF-36-GH, M (IQR)	50 (42)	47 (42)	0.299
SF-36-VT, M (IQR)	80 (40)	60 (40)	0.006*
SF-36-SF, M (IQR)	62.5 (25)	50 (25)	0.034*
SF-36-RE, M (IQR)	0 (66)	0 (33)	0.081
SF-36-MH, M (IQR)	68 (32)	64 (28)	0.042*
SF-36-HT, M (IQR)	75 (25)	75 (25)	0.793
Preoperative JOA, M (IQR)	13.5 (1.5)	11.5 (3.5)	<0.001*
Gender (male, n, %)	183 (60.4)	187 (50.7)	0.012*
Operation			
Anterior (n, %)	151 (49.8)	189 (51.2)	0.725
Posterior (n, %)	131 (43.2)	150 (40.7)	
Anterior + posterior (n, %)	21 (6.9)	30 (8.1)	
Diagnosis			
CSM (n, %)	199 (65.7)	242 (65.7)	0.991
OPLL (n, %)	7 (2.3)	8 (2.2)	
CSM + OPLL (n, %)	97 (32.0)	119 (32.2)	
High signals on T2WI (n, %)			
None (n, %)	83 (27.4)	117 (31.7)	0.176
Single-level (n, %)	176 (58.1)	214 (58.0)	
Multiple-level (n, %)	44 (14.5)	38 (10.3)	
Drinking alcohol (n, %)	43 (14.2)	19 (5.1)	<0.001*
Smoking history (n, %)	65 (21.5)	46 (12.5)	0.002*
Symptom			
Numbness in upper limbs (n, %)	245 (80.9)	293 (79.4)	0.639
Numbness in lower limbs (n, %)	104 (34.3)	143 (38.8)	0.236
Trunk numbness (n, %)	6 (2.0)	20 (5.4)	0.021*
Weakness in upper limbs (n, %)	53 (17.5)	77 (20.9)	0.270
Weakness in lower limbs (n, %)	166 (54.8)	204 (55.3)	0.897
Pain in upper limbs (n, %)	56 (18.5)	67 (18.2)	0.914
Pain in lower limbs (n, %)	19 (6.3)	15 (4.1)	0.194
Trunk pain (n, %)	22 (7.3)	52 (14.1)	0.005*
Shoulder pain (n, %)	55 (18.2)	79 (21.4)	0.293
Neck pain (n, %)	118 (38.9)	153 (41.5)	0.508
Band-like sensation (n, %)	22 (7.3%)	44 (11.9%)	0.043*
Sensation of walking on cotton wool (n, %)	164 (54.1%)	217 (58.8)	0.223
Fine motor skills (n, %)	59 (19.5)	76 (20.6)	0.717
Symptoms of lumbar spinal stenosis (n, %)	29 (9.6)	46 (12.5)	0.236
Autonomic symptoms (n, %)	59 (19.5)	91 (24.7)	0.108
Bowel and bladder function (n, %)	4 (1.3)	6 (1.6)	1.000
Muscle atrophy (n, %)	36 (11.9)	37 (10.0)	0.442
Neck tenderness (n, %)	157 (51.8)	230 (62.3)	0.006*
Upper limb muscle strength (n, %)	148 (48.8)	197 (53.4)	0.241
Lower limb muscle strength (n, %)	83 (27.4)	114 (30.9)	0.321
Abdominal reflex (n, %)	12 (4.0)	15 (4.1)	0.945
Cremasteric reflex (n, %)	4 (1.3)	2 (0.5)	0.417
Anal reflex (n, %)	1 (0.3)	0 (0)	0.451
Upper limb reflex (n, %)	11 (3.6)	15 (4.1)	0.771
Lower limb reflex (n, %)	13 (4.3)	13 (3.5)	0.608
Pathological sign			
Hoffmann sign (n, %)	262 (86.5)	309 (83.7)	0.325
Babinski sign (n, %)	108 (35.6)	160 (43.4)	0.042*
With comorbidities (n, %)	94 (31.0)	120 (32.5)	0.678
Brachial plexus nerve stretch test (n, %)	50 (16.5)	69 (18.7)	0.458
Intervertebral foramen compression test (n, %)	35 (11.6)	47 (12.7)	0.640

IQR, interquartile range.

*P < 0.05.

### 3.2 Model generation

**FIGURE 2:**
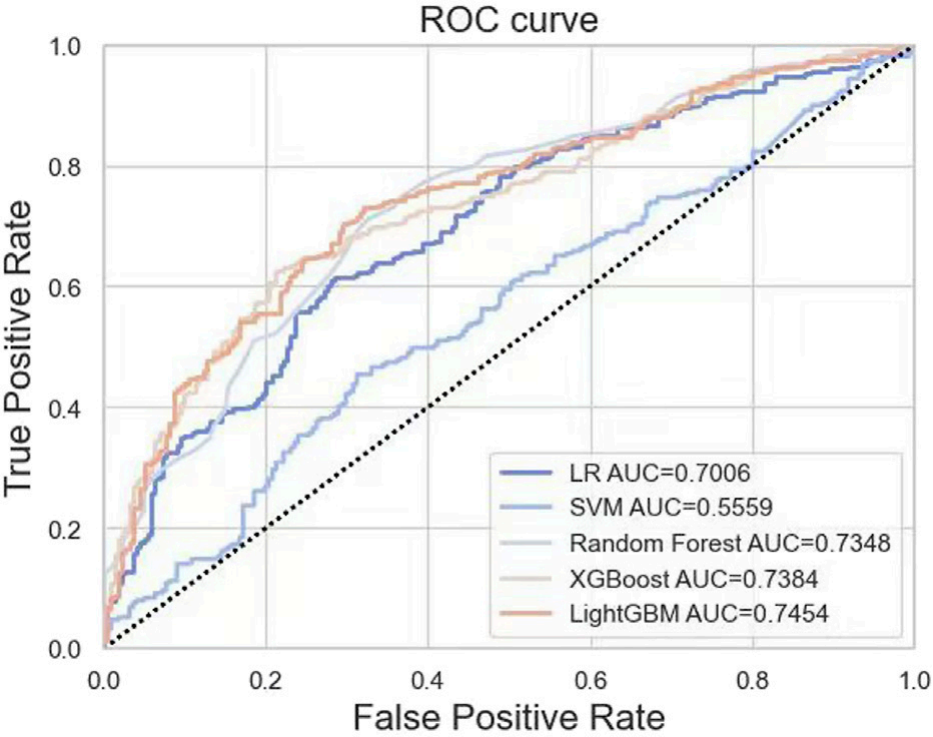
ROC curves generated by 3-fold cross-validation algorithms in the training set.

**FIGURE 3:**
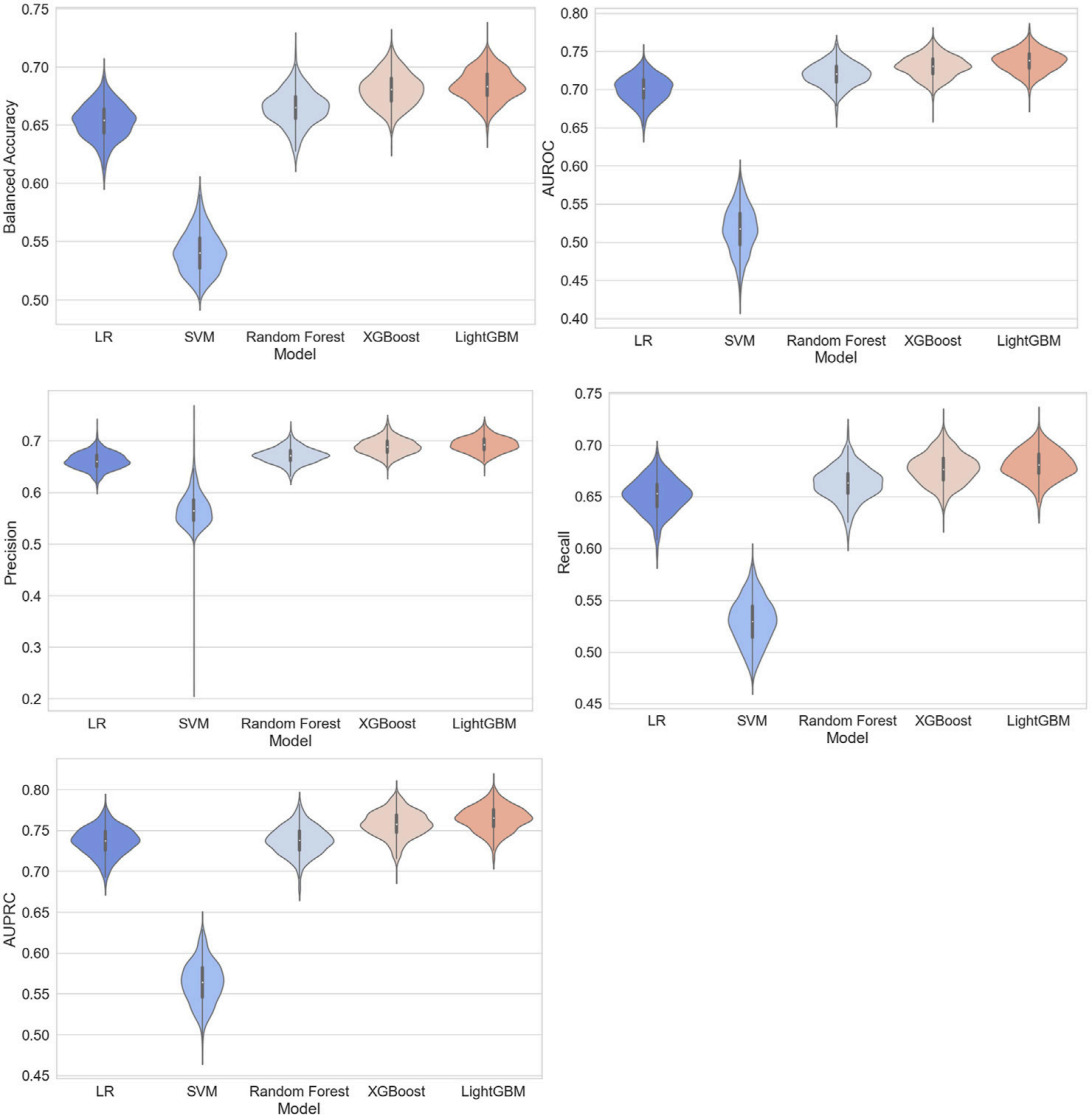
Comparisons of balanced accuracy, weighted area under the precision*–*recall curve (AUPRC), weighted precision, weighted recall, and AUPRC of the five models in the training set.

**FIGURE 4:**
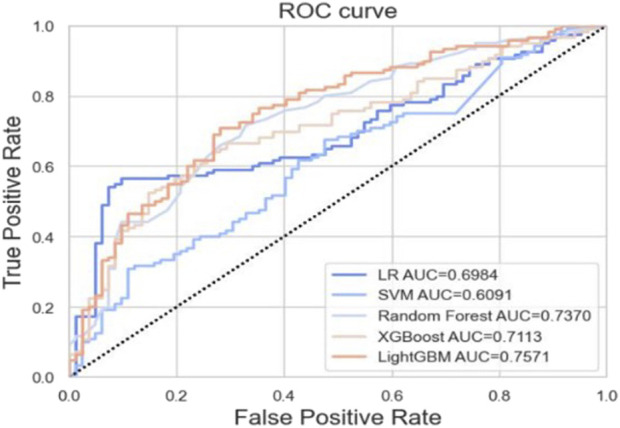
ROC curves generated by 3-fold cross-validation algorithms in the testing set.

Based on the training set, we generated five models (LR, SVM, RF, XGBoost, and LightGBM). The ROC curves and AUROC values produced by the three-fold cross-validation algorithms are presented in Figure 2. Figure 3 presents the comparisons of the balanced accuracy, weighted precision, weighted recall, AUPRC, and AUROC values in the training set. The decision tree algorithms (RF, XGBoost, and LightGBM) demonstrated superior performance among the five selected machine learning models. The LightGBM model achieved the highest AUROC value of 0.745. We used the testing set to evaluate the five trained models. The AUROC values of the training and testing sets are summarized in Table 2. The LightGBM model demonstrated the best performance in training and testing sets, with a slight decrease in the AUROC value, indicating no overfitting (Figure 4).

**TABLE 2:** AUROC values were generated by 3-fold cross-validation algorithms in the training and testing sets.

Model	LR	SVM	RF	XGBoost	LightGBM
Training set AUROC*	0.701 [0.664, 0.732]	0.556 [0.457, 0.690]	0.735 [0.690, 0.749]	0.738 [0.702, 0.758]	0.763 [0.708,0.764]
Testing set AUROC*	0.698 [0.65, 0.774]	0.609 [0.352, 0.660]	0.737 [0.670, 0.791]	0.711 [0.669,0.791]	0.757 [0.702, 0.816]

*The values are given as the mean and the 95% confidence intervals.

**FIGURE 5:**
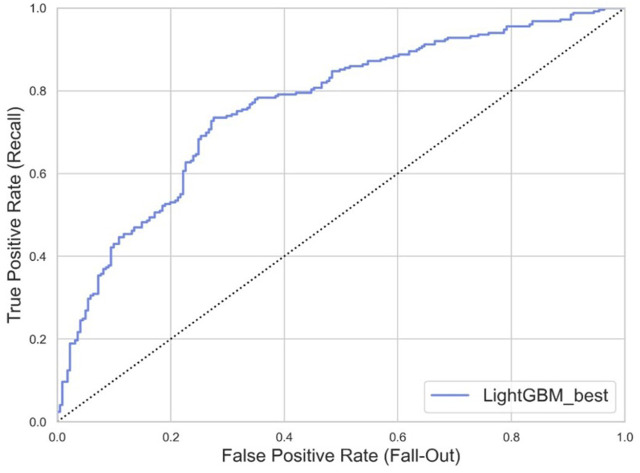
ROC curve of the tuned LightGBM model. The area under curve (AUROC) value was 0.763.

The grid search strategy was used to optimize the hyperparameters of LightGBM. We tuned six hyperparameters, including “max_depth,” “num_leaves,” “subsample,” “colsample_bytree,” “reg_alpha,” and “reg_lambda.” The search spaces and best values are provided in Table 3. The AUROC value of the tuned LightGBM model increased to 0.763 in the training set (Figure 5).

**TABLE 3:** Search spaces and best values of hyperparameters in the LightGBM model.

Hyperparameter	Search space	Best value
max_depth	[3,4,5]	3
num_leaves	[5, 6, 7, 12, 13, 14, 15, 28, 29, 30, 31]	5
subsample	[0.8, 0.9, 1.0]	0.8
colsample_bytree	[0.8, 0.9, 1.0]	1.0
reg_alpha	[0,10,100,1000]	0
reg_lambda	[0,10,100,1000]	10

### 3.3 Feature importance

**FIGURE 6:**
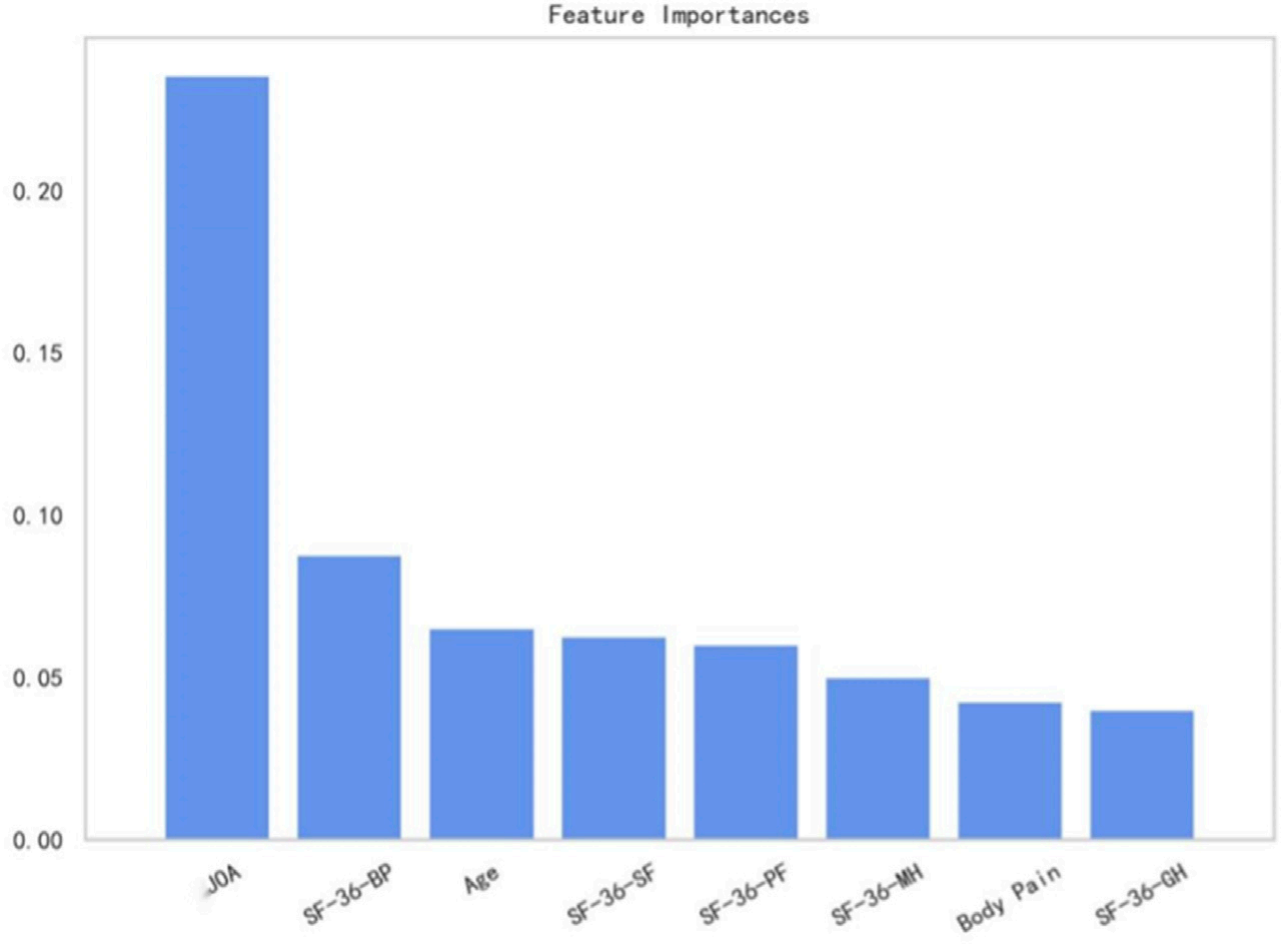
Top eight most essential predictors for the prediction of outcomes.

**FIGURE 7:**
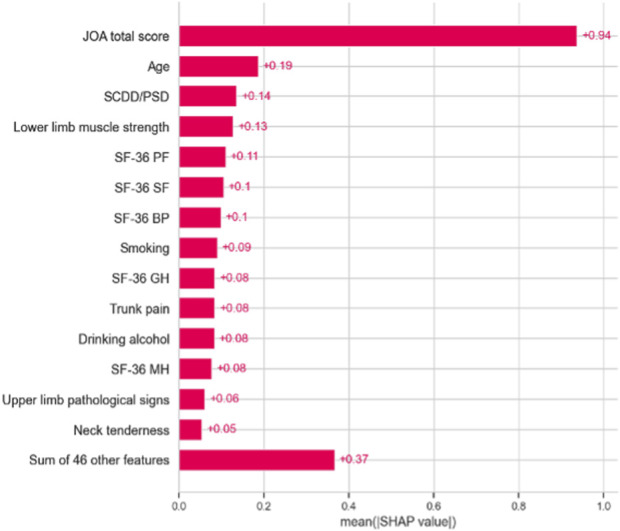
Bar plot of the SHAP value of the predictors in the model.

**FIGURE 8:**
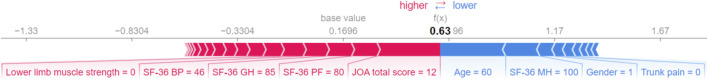
Single-sample SHAP force plot. Blue indicates that the feature has a negative effect on the prediction (arrow to the left, SHAP value decreases), and red indicates that the feature has a positive effect on the prediction (arrow to the right, SHAP value increases). On the number line, 0.1696 is the base SHAP value, which is the average predicted by the model. In this sample, the total JOA score = 12 had the most significant positive effect and age = 60 years had the most significant negative effect, resulting in a SHAP value of 0.63 for this patient.

We evaluated the feature importance of the LightGBM model. Preoperative JOA scores, SF-36-BP, age, SF-36-SF, SF-36-PF, SF-36-MH, body pain, and SF-36-GH are the eight most critical predictors for outcome prediction (Figure 6). We selected the top 10 features based on feature importance, and the SHAP values were calculated to evaluate the importance of features at the individual level (Figure 7). A single-sample SHAP force plot for a patient is shown in Figure 8.

### 3.4 Feature-reduced model

**FIGURE 9:**
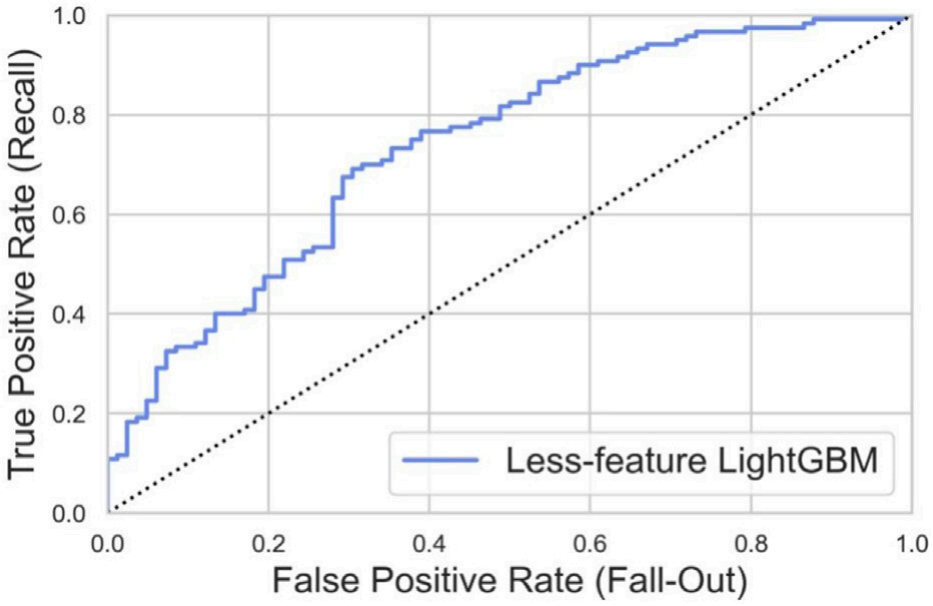
ROC curve of the feature-reduced model in the testing set with an area under curve (AUROC) value of 0.734.

According to the feature importance and the accessibility of input data, we chose age, sex, preoperative JOA scores, and preoperative SF-36 scores as features to build the feature-reduced model. We applied the same training set to construct the feature-reduced model. Then, the grid-search algorithm was used to optimize the hyperparameter with the same search spaces. The best value is presented in Table 4. The feature-reduced model was also tested with the same dataset. The ROC curve in the testing set is shown in Figure 9. The feature-reduced LightGBM model had an AUROC value of 0.734, which showed almost the same performance as the initial model (Figure 9).

**TABLE 4:** Best value of the hyperparameters in the feature-reduced model.

Hyperparameter	Parameter space	Best Value
max_depth	[3,4,5]	4
num_leaves	[5, 6, 7, 12, 13, 14, 15, 28, 29, 30, 31]	5
subsample	[0.8, 0.9, 1.0]	0.8
colsample_bytree	[0.8, 0.9, 1.0]	0.9
reg_alpha	[0,10,100,1000]	0
reg_lambda	[0,10,100,1000]	0

### 3.5 External validation

**FIGURE 10:**
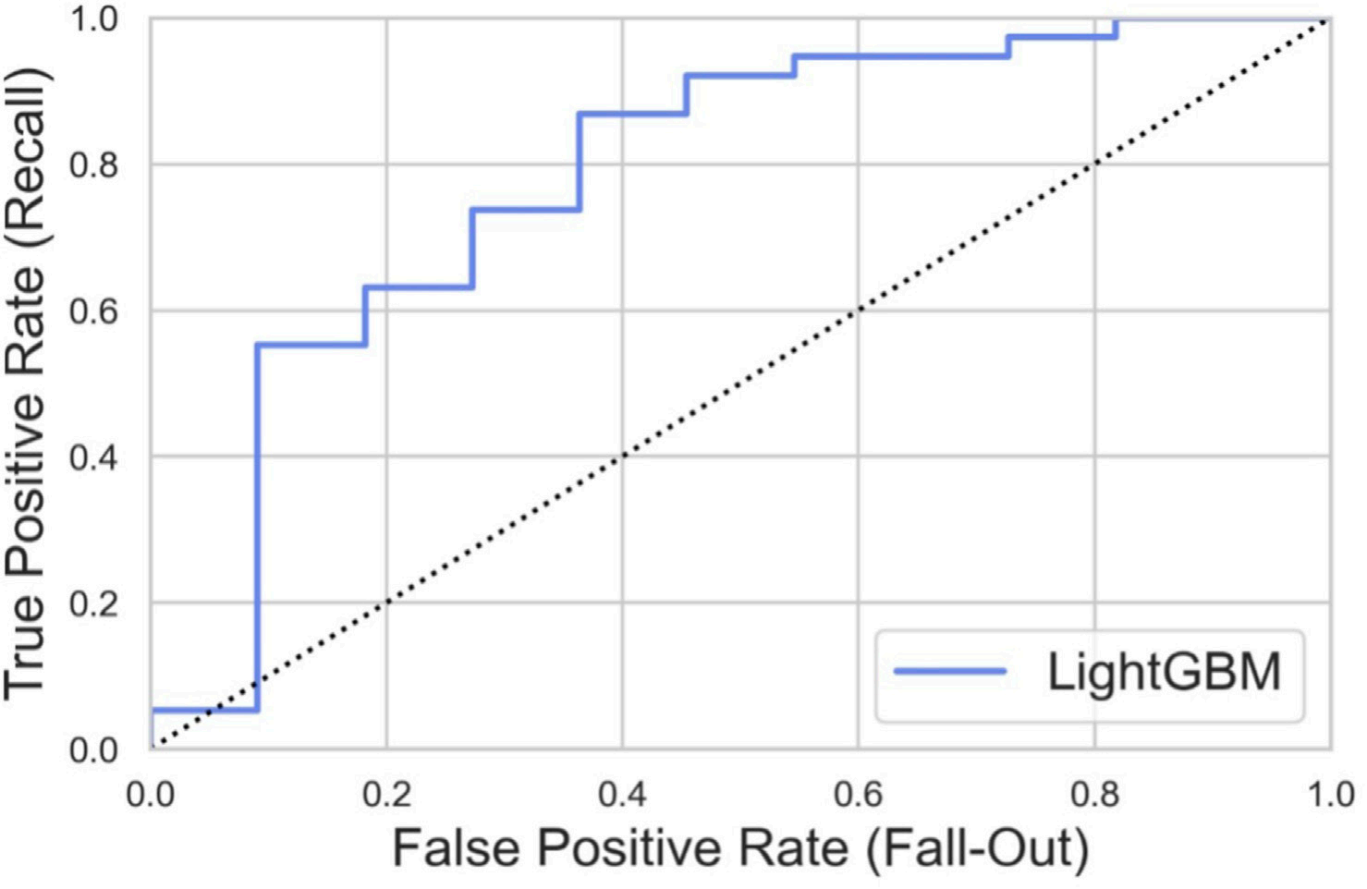
ROC curve of the feature-reduced model in the external dataset with an area under curve (AUROC) value of 0.785.

The external dataset included 31 patients from West China Hospital and 27 patients from Shanghai Changzheng Hospital. To generate the external testing set, we extracted the data according to the reduced features (age, sex, preoperative JOA scores, and preoperative SF-36 scores). The outcome measure was whether patients achieved the MCID of the JOA score at 3–6 months after the DCM decompression surgery. The ROC curve is presented in Figure 10, with an AUROC value of 0.785. This indicated that our model also performed well in extrapolation.

## 4 Discussion

Surgical decompression is recommended for the treatment of DCM and has shown long-term improvement in patients. However, the degree of patient recovery can vary widely (Gulati et al., 2021). In previous studies, several machine learning models have been developed to predict improvements in neurological function using the preoperative clinical characteristics of patients with DCM (Khan et al., 2021; Merali et al., 2019; Song et al., 2024). In this study, we applied a natural language processing algorithm to extract symptom data from unstructured medical history records, which could include more features than previous studies. We compared the performance of each model and identified that the LightGBM model demonstrated the best predictive performance. To the best of our knowledge, we were the first to build a feature-reduced model based on LightGBM to predict surgery outcomes for DCM patients, which showed good performance.

We built machine learning models using five classic algorithms. The decision tree algorithms (RF, XGBoost, and LightGBM) showed better performance with an AUROC value of more than 0.7, and the LightGBM model showed the best performance. Decision tree algorithms correlate features with outcomes and have been effectively utilized across various learning disciplines. At each node or branch point, training examples are partitioned based on the value of a particular feature (Shah et al., 2023). Maki et al. reported that XGBoost showed the highest AUROC value (0.72) for predicting the MCID of the JOA score in patients with cervical ossification of the posterior longitudinal ligament (OPLL) 1 year after surgical treatment, whereas RF demonstrated the highest AUROC value (0.75) for predicting MCID at 2 years (Maki et al., 2021). Merali et al. (2019) reported that the best-performing predictive model used a random forest structure with an average area under the curve (AUROC) value of 0.70 to predict postoperative MCID of SF-6D and mJOA score in DCM patients at 6-, 12-, and 24-month follow-up. Our results showed that decision tree algorithms have a good predictive ability for surgical outcomes in DCM patients, which was consistent with prior studies.

The feature importance of the LightGBM model was calculated, and the preoperative JOA scores, SF-36-BP, age, SF-36-SF, SF-36-PF, SF-36-MH, body pain, and SF-36-GH are the top eight most crucial predictors for the prediction of neurological outcomes. Previous studies have demonstrated that preoperative clinical features of patients with DCM were associated with neurological function improvement after surgery. It was reported that gender, age, and preoperative functional scores were related to the surgical prognosis of patients with DCM (Tetreault et al., 2018; Nakashima et al., 2012). We found that body pain correlated to the trunk sensation and was a crucial predictor of surgery outcome. Previous studies showed that positive pathologic signs of lower limbs, finger numbness, and hyperreflexia are associated with unsatisfactory improvement of nerve function measured by JOA after surgery (Tetreault et al., 2013; Tetreault et al., 2016; Tetreault L. et al., 2015). The body pain could also be a sign of more serious neurological impairment and predict worse improvement.

We demonstrated that patients with lower preoperative JOA scores were more likely to achieve MCID in their recovery of neurological function at 3–6 months after surgery. This may be because higher preoperative JOA scores have less room for improvement due to ceiling effects, similar to previous studies for the prediction of MCID (Khan et al., 2021; Maki et al., 2021). Patients with lower preoperative JOA scores could be more likely to benefit from surgery. This insight is crucial for understanding the prognosis of DCM patients after surgery.

We built a feature-reduced model and showed that the feature-reduced model could get the same prediction accuracy by only paying attention to age, sex, preoperative JOA scores, and preoperative SF-36 scores. As far as we know, this is the first study to build a feature-reduced machine learning model to predict DCM patient outcomes after surgical treatment. However, the feature-reduced LightGBM model showed slightly worse performance in the testing set, which might be attributed to fewer features. Reducing the number of features could enhance the model’s generalizability. In external validation using an independent patient cohort from two hospitals, our model performed well with an AUROC value of 0.785. This indicated that developing a feature-reduced model could be an effective strategy for the generalization of the prediction model of DCM patient outcomes.

Our study still has some limitations. First, our follow-up data are limited to the short-term period of 3–6 months. Second, preoperative clinical variables only include intramedullary high signals on T2WI MRI. More preoperative imaging parameters of patients with DCM may improve the predictive efficacy of the model (Naruse et al., 2009; Hamburger et al., 1997). Third, the amount of data we have is not particularly large. Although machine learning offers a powerful way to build complex models and generate predictions, compared to traditional statistical methods, machine learning models require relatively large datasets to achieve optimal performance.

## 5 Conclusion

We established models to predict postoperative neurological outcomes based on machine learning. The LightGBM model presented the best predictive power with an AUROC value of 0.745. Feature importance analysis showed that age, preoperative JOA score, SF-36-PF, SF-36-GH, and SF-36-BP were crucial factors for prediction. The feature-reduced model could achieve almost the same prediction accuracy by only paying attention to age, sex, preoperative JOA scores, and preoperative SF-36 scores, making it more practical for clinical application. Machine learning could help spine surgeons make more precise predictions of DCM patient outcomes after cervical decompression surgery.

## Data Availability

The raw data supporting the conclusions of this article will be made available by the authors, without undue reservation.
