# Supplementary material for: Predicting postoperative neurological outcomes of degenerative cervical myelopathy based on machine learning
Source: Front Bioeng Biotechnol. 2025 Mar 4;13:1529545. doi: 10.3389/fbioe.2025.1529545 (PMC11913819; doi:10.3389/fbioe.2025.1529545)
Supplement: Supplementary file 1 [file DataSheet1.docx]

Balanced accuracy: Defined as the arithmetic mean of sensitivity (true positive rate) and specificity (true negative rate). *Mosley, Lawrence. "A balanced approach to the multi-class imbalance problem." (2013)*

Weighted precision/recall: Precision represents the proportion of predicted positive instances that are truly positive (TP/(TP+FP)), while recall represents the proportion of actual positives correctly identified (TP/(TP+FN)). In the context of weighted precision and recall, these metrics are calculated separately for each class (positive or negative in our study). Then, a weighted average of the precision and recall values is computed, where the weights correspond to the number of samples in each class. This approach effectively addresses class imbalance by weighing the prevalence of classes.

Weighted AUPRC: By computing a weighted precision and weighted recall at every threshold of the model, one can plot a precision-recall curve. AUPRC is the area under the precision-recall curve. *Boyd, K., Eng, K. H., & Page, C. D. (2013). Area under the precision-recall curve: point estimates and confidence intervals. In Machine Learning and Knowledge Discovery in Databases: European Conference, ECML PKDD 2013, Prague, Czech Republic, September 23-27, 2013, Proceedings, Part III 13 (pp. 451-466). Springer Berlin Heidelberg.*

AUROC: By computing a true positive rate and false positive rate at every threshold of the model, one can plot a receiver operating characteristic (ROC) curve. AUROC is the area under the ROC curve. *Hanley JA, McNeil BJ. A method of comparing the areas under receiver operating characteristic curves derived from the same cases. Radiology. 1983 Sep;148(3):839-43. doi: 10.1148/radiology.148.3.6878708. PMID: 6878708.*

SHAP (SHapley Additive exPlanations)

SHAP is a game-theory-based method for interpreting machine learning models. The SHAP value of a feature is calculated based on the marginal contribution of the feature across all possible feature subsets and then average those contributions to yield that feature’s overall impact on the prediction. *Scott, M., & Su-In, L. (2017). A unified approach to interpreting model predictions. Advances in neural information processing systems, 30, 4765-4774.*
